# Radiomics predict the WHO/ISUP nuclear grade and survival in clear cell renal cell carcinoma

**DOI:** 10.1186/s13244-024-01739-z

**Published:** 2024-07-12

**Authors:** Xiaoxia Li, Jinglai Lin, Hongliang Qi, Chenchen Dai, Yi Guo, Dengqiang Lin, Jianjun Zhou

**Affiliations:** 1 Department of Radiology, Zhongshan Hospital (Xiamen), Fudan University, Xiamen, 361015 China; 2 Department of Urology, Zhongshan Hospital (Xiamen), Fudan University, Xiamen, 361015 China; 3 Department of Clinical Engineering, Southern Medical University, Nanfang Hospital, Guangzhou, 510515 China; 4 Department of Radiology, Zhongshan Hospital, Fudan University, No 180, Fenglin Road, Xuhui District, Shanghai, 200032 China; 5 Xiamen Municipal Clinical Research Center for Medical Imaging, Xiamen, 361015 China; 6 Fujian Province Key Clinical Specialty for Medical Imaging, Xiamen, 361015 China; 7 Xiamen Key Laboratory of Clinical Transformation of Imaging Big Data and Artificial Intelligence, Xiamen, 361015 China

**Keywords:** Renal cell carcinoma, WHO/ISUP grade, Radiomics, Overall survival

## Abstract

**Objectives:**

This study aimed to assess the predictive value of radiomics derived from intratumoral and peritumoral regions and to develop a radiomics nomogram to predict preoperative nuclear grade and overall survival (OS) in patients with clear cell renal cell carcinoma (ccRCC).

**Methods:**

The study included 395 patients with ccRCC from our institution. The patients in Center A (anonymous) institution were randomly divided into a training cohort (*n* = 284) and an internal validation cohort (*n* = 71). An external validation cohort comprising 40 patients from Center B also was included. Computed tomography (CT) radiomics features were extracted from the internal area of the tumor (IAT) and IAT combined peritumoral areas of the tumor at 3 mm (PAT 3 mm) and 5 mm (PAT 5 mm). Independent predictors from both clinical and radiomics scores (Radscore) were used to construct a radiomics nomogram. Kaplan–Meier analysis with a log-rank test was performed to evaluate the correlation between factors and OS.

**Results:**

The PAT 5-mm radiomics model (RM) exhibited exceptional predictive capability for grading, achieving an area under the curves of 0.80, 0.80, and 0.90 in the training, internal validation, and external validation cohorts. The nomogram and RM gained from the PAT 5-mm region were more clinically useful than the clinical model. The association between OS and predicted nuclear grade derived from the PAT 5-mm Radscore and the nomogram-predicted score was statistically significant (*p* < 0.05).

**Conclusion:**

The CT-based radiomics and nomograms showed valuable predictive capabilities for the World Health Organization/International Society of Urological Pathology grade and OS in patients with ccRCC.

**Critical relevance statement:**

The intratumoral and peritumoral radiomics are feasible and promising to predict nuclear grade and overall survival in patients with clear cell renal cell carcinoma, which can contribute to the development of personalized preoperative treatment strategies.

**Key Points:**

- The multi-regional radiomics features are associated with clear cell renal cell carcinoma (ccRCC) grading and prognosis.
- The combination of intratumoral and peritumoral 5 mm regional features demonstrated superior predictive performance for grading.
- The nomogram and radiomics models have a broad range of clinical applications.

**Graphical Abstract:**

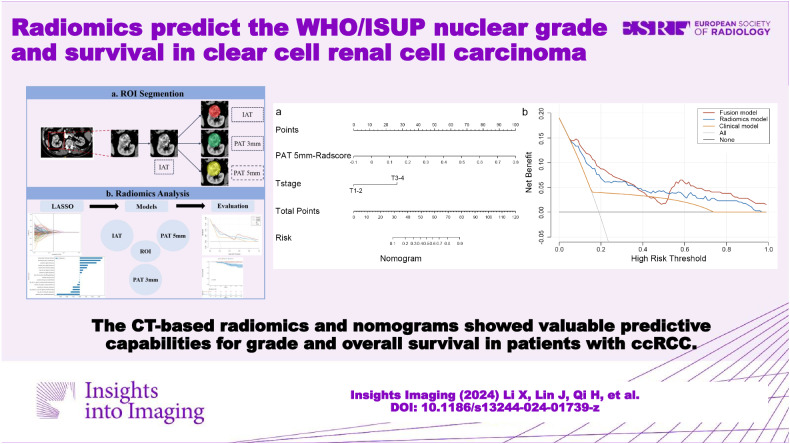

## Introduction

Renal cell carcinoma (RCC) is the most common type of primary kidney cancer, with clear cell RCC (ccRCC) accounting for the majority of cases [1]. The pathological nuclear grade is an important prognostic factor for patients with ccRCC [2]. In 2016, the widely accepted classification system for ccRCC was established by The World Health Organization/International Society of Urological Pathology (WHO/ISUP) criteria. This system categorizes ccRCC into four grades, identifying grades I and II as low-grade and grades III and IV as high-grade [3]. Low-grade ccRCC may be treated with less aggressive options, whereas high-grade ccRCC may require more intensive treatment [4]. This categorization serves to decrease discrepancies between observers, enhance reproducibility, and save time without affecting the ability to predict cancer-specific mortality [5, 6]. Assessment of pathological grading prior to surgery commonly relies on biopsy. However, due to the high spatiotemporal heterogeneity of ccRCC, biopsies may represent only a portion of the lesions that may contribute to selection and may not adequately reflect the nuclear grade of the entire tumor. This invasive procedure also suffers from low reproducibility and a high rate of complications [7]. Therefore, the development of a non-invasive method for predicting ccRCC grading before surgery would greatly benefit clinical practice.

There is a promising future for the application of radiomics in the prediction of malignant tumors, tumor histopathology, tumor grading, and molecular characteristics [8]. Many radiomics investigations have concentrated on predicting the nuclear grading to assess tumor invasiveness [9–16]. In a study by Cui et al, multiparametric magnetic resonance imaging and multiphase CT-based machine-learning models were created from the radiomics features of a group of 460 patients diagnosed with ccRCC. These models achieved an accuracy of 73% and 79% when distinguishing between high-grade and low-grade ccRCC [13]. Another study demonstrated that a radiomics signature consisting of 20 features displayed excellent performance in differentiating grades in a multi-institutional cohort of 258 patients, with an area under the curve (AUC) of 0.846 in the validation sets [17]. However, these models solely relied on intratumoral radiomics and did not incorporate peritumoral radiomics. Peritumoral and multi-regional radiomics are increasingly used in tumor diagnosis, grading, and prognosis in other cancers [18–21], but their application in RCC remains relatively rare. Recent research suggests that peritumoral radiomics has a significant role in ccRCC grading [22]. Additionally, most studies have failed to consider the integration of clinical risk factors. It is plausible to hypothesize that models capable of accurately predicting grading could also exhibit strong performance in predicting survival given the close association between grading and prognosis. Unfortunately, most studies have primarily focused on the predictive aspect of grading while disregarding its correlation with prognosis. Thus, the development of a suitable model that not only accurately predicts ccRCC grading but also elucidates the underlying survival mechanisms associated with prognosis presents a significant challenge [23].

This study aimed to evaluate the predictive value of radiomics extracted from intratumoral and peritumoral regions in patients with ccRCC and develop a CT radiomics nomogram for predicting the WHO/ISUP grade. Additionally, we investigated whether the constructed grading prediction model was associated with the overall survival (OS) of ccRCC.

## Materials and methods

### Patients

**Fig. 1:**
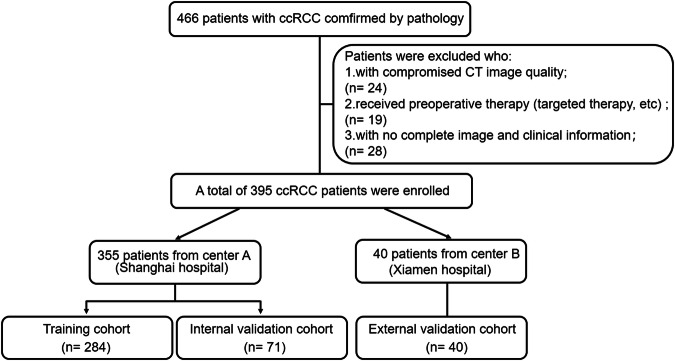
Flow chart showing the pathway of patient exclusion

This retrospective study complied with the ethical guidelines of the Declaration of Helsinki and was approved by the ethics committee of our hospital, and the requirement for informed consent was waived. We collected data from the hospital’s medical records, including 355 patients with ccRCC who underwent radical or partial nephrectomies between January 2015 and December 2018. The inclusion and exclusion criteria are presented in Appendix S1 and Fig. 1. Additionally, we obtained 40 patients from our branch hospital for external validation in the same inclusion and exclusion criteria. The collected clinical data are presented in Table 1.

**Table 1:** Characteristics of ccRCC patients in the training set, internal validation set, and external validation set

	Training set (*n* = 284)			Internal validation set (*n* = 71)			External validation set (*n* = 40)		
	Low grade	High grade	*p*-value	Low grade	High grade	*p*-value	Low grade	High grade	*p*-value
Age (year)	58.3 ± 11.8	61.2 ± 7.9	0.15	58.4 ± 11.6	65.5 ± 6.4	0.02^a^	57.2 ± 11.2	62.1 ± 11.8	0.19
R-tumor size (cm)	4.1 ± 2.0	5.5 ± 2.1	< 0.001^a^	3.9 ± 2.0	5.8 ± 2.1	0.001^a^	3.5 ± 1.4	5.8 ± 2.4	0.002^a^
Sex			0.49			0.13			0.35
Female	79 (33)	13 (27)		18 (32)	1 (7)		7 (26)	1 (8)	
Male	157 (67)	35 (73)		39 (68)	13 (93)		20 (74)	12 (92)	
P-T stage			< 0.001^a^			0.04^a^			0.05
T1-2	230 (97)	36 (75)		57 (100)	12 (86)		27 (100)	10 (77)	
T3-4	6 (3)	12 (25)		Null	2 (14)		Null	3 (23)	
ECOG-PS									
0	198 (84)	42 (87)	0.53	45 (79)	10 (71)	0.54	25 (93)	12 (92)	0.974
≥ 1	38 (16)	6 (13)		12 (21)	4 (29)		2 (7)	1 (8)	

Quantitative variables were expressed as mean ± standard deviation for age and tumor size, and analyzed by Student’s *t*-test or Mann–Whiney *U*-test. For qualitative variables for sex and T stage, percentages or frequencies were calculated, and a *χ*^2^ test. R-tumor size was based on radiology and P-T stage was based on pathology. *ECOG-PS* Eastern Cooperative Oncology Group Performance Status

^a^ Significant results

### Image acquisition and tumor segmentation

**Fig. 2:**
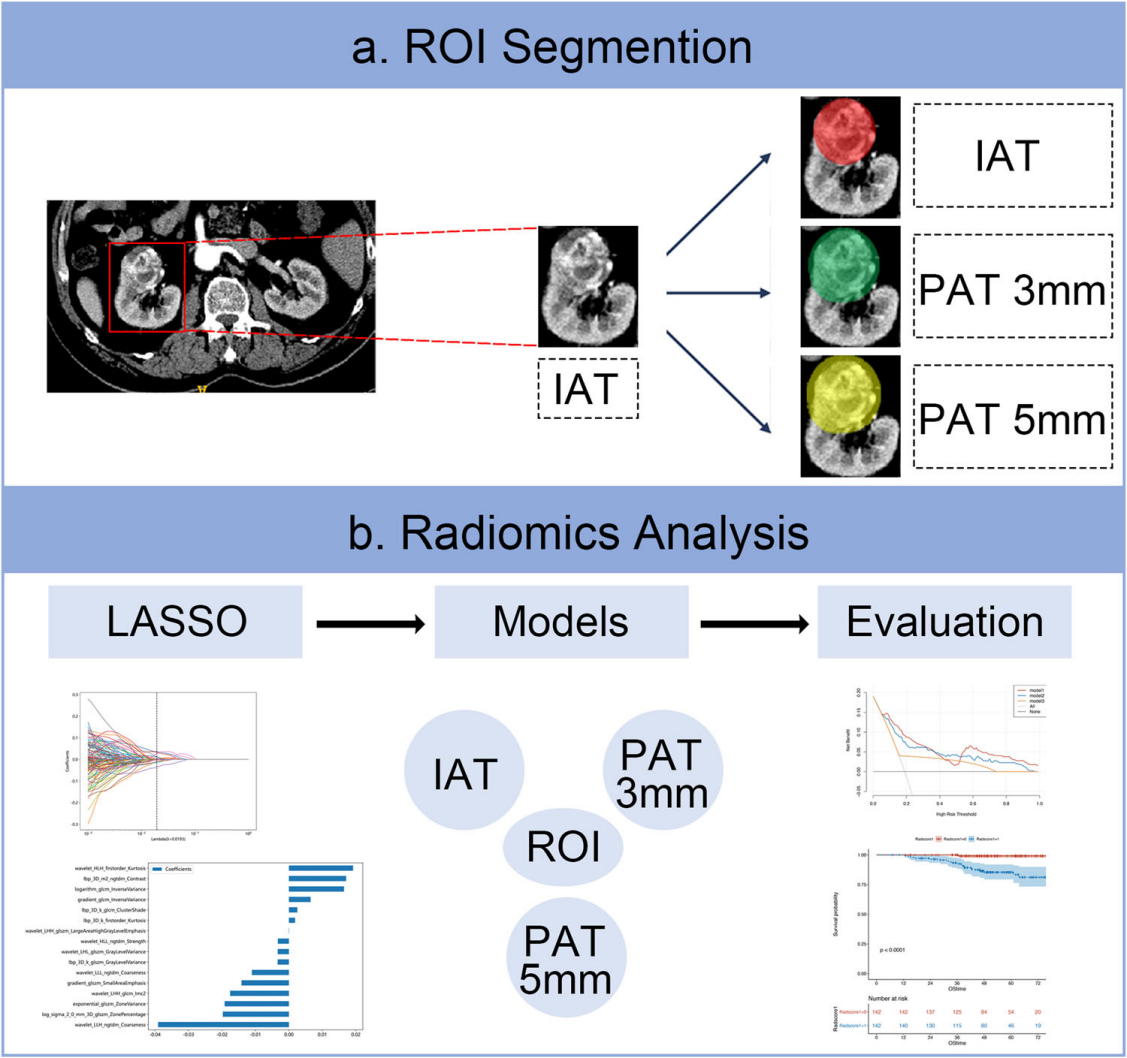
Study workflow. **a** ROI segmentation, **b** radiomics analysis

All patients undergo preoperative CT-enhanced examination with 16-detector and 64-detector. Arterial-phase CT images of the patients with ccRCC were analyzed. The detailed CT parameters are listed in Appendix S2. The details about tumor segmentation are illustrated in Appendix S3 and Fig. 2.

### Radiomics feature selection and reproducibility analysis

Before conducting feature extraction, the CT images went through preprocessing procedures involving resampling and standardization to ensure that the results could be replicated. Pyradiomics v3.0.1 package (https://pypi.org/project/pyradiomics/) was performed to extract radiomics features from the regions of interests (ROIs). The details about the extracted features are illustrated in Appendix S4. Initially, the *z*-score method was used to standardize these radiomics features. The inter- and intra-observer and correlation coefficients (ICCs) were calculated for reproducibility analysis in Appendix S5.

### Feature selection and radiomics model construction

After selecting the remaining features with an ICC > 0.75, the statistical significance of these features was determined by performing the student’s *t*-test or the Mann–Whitney *U*-test. Only the features with values of *p* < 0.05 were considered. Spearman’s correlation coefficient was then used to evaluate the correlations between features that exhibited high repeatability, and any two features with a correlation coefficient > 0.9 were retained. Next, the least absolute shrinkage and selection operator (LASSO) algorithm was used to create the dataset’s signature. A 10-fold cross-validation with minimum criteria was used to find the optimal value of *λ*, in which the value of *λ* that resulted in the lowest cross-validation error was chosen. The features with nonzero coefficients from the LASSO algorithm were used to construct radiomics models (RMs) for predicting grading and combined into a radiomics score (Radscore). To assess the discrimination performance of the radiomics signature, the Wilcoxon test was used to compare the difference in the Radscores between the low-grade and high-grade groups. The performance of the RMs in predicting grading was further evaluated using the area under the receiver operating characteristic curve in the training set, internal validation set, and external validation.

### Nomogram construction and survival analysis

To create a clinical model (CM), univariable and multivariable logistic regression analyses were performed to identify factors that independently predicted nuclear grade. These analyses included variables, such as age, sex, tumor size, and T stage. A fusion model (FM) was constructed that was based on the Radscore of the best RM and independent clinical predictors. Then, ROC curves were used to evaluate the performances of the CM, RM, and FM models in the three sets. To assess the clinical usefulness of the models, decision curve analysis (DCA) was performed. The Kaplan–Meier method was used to analyze survival outcomes in different groups stratified by nomogram-predicted low and high risk and to create corresponding survival curves. Additionally, the log-rank test was conducted to evaluate the statistical significance of any differences observed between these groups.

### Statistical analysis

Student’s *t*-test, or Mann–Whitney *U*-test, chi-square, or Fisher’s exact test was used to analyze the difference between low- and high-grade groups. To identify independent predictors for grading ccRCC patients, we performed both univariable and multivariable logistic regression analyses. For statistical significance, we considered two-sided *p*-values of < 0.05. All statistical analyses were performed in SPSS v.20.0, R software (version 4.2.0), and Pytorch v.1.8.0.

## Results

### Patients characteristics

The characteristics of the ccRCC patients, including the distribution of patients in the training set, internal validation set, and external validation set, are presented in Table 1. There were significant differences in age, radiological tumor size, and pathological T stage based on CT between low-grade and high-grade patients in the training set. Multivariable logistic regression analysis identified the T stage as an independent predictor of ccRCC grading (Table 2). This valuable information was used to establish the CM.

**Table 2:** Univariable and multivariable logistic regression analysis of factors in the training

	Univariable regression		Multivariable regression	
	OR (95% CI)	*p*-value	OR (95% CI)	*p*-value
Age, years	1.005 (1.002–1.007)	0.012^a^		
Sex (male vs. female)	1.073 (1.000–1.153)	0.105		
R-tumor size	1.051 (1.036–1.067)	< 0.001^a^		
P-T stage (T1-2 vs. T3-4)	1.745 (1.523–2.000)	< 0.001^a^	1.552 (1.362–1.768)	< 0.001^a^
ECOG-PS	0.744 (0.269–1.759)	0.531		
RM_5_-Radscore	4.019 (2.995–5.392)	< 0.001^a^	4.094 (2.578–6.501)	< 0.001^a^

R-tumor size was based on radiology and P-T stage was based on pathology. *ECOG-PS* Eastern Cooperative Oncology Group Performance Status

*RM*_*5*_ radiomics model based on intratumoral and peritumoral 5 mm regional features

^a^ Significant results

### Feature selection

**Fig. 3:**
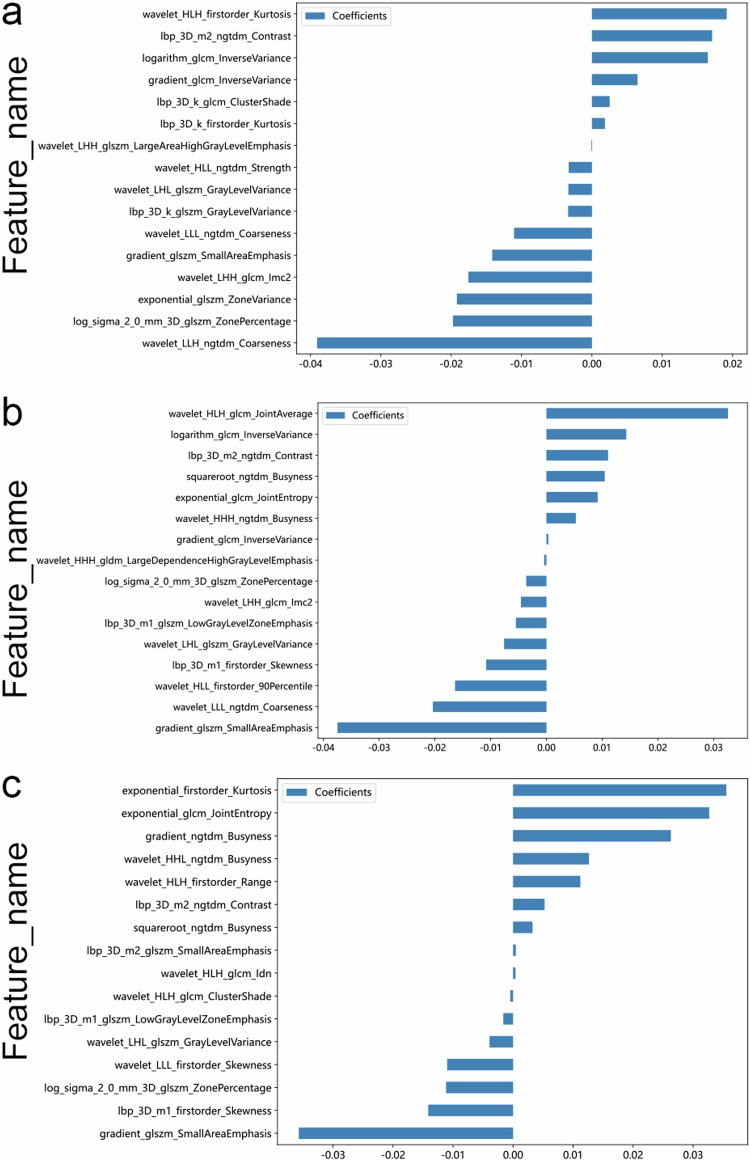
The retained radiomics features and corresponding coefficients of different models after performing LASSO regression analysis. **a** IAT model, **b** PAT 3-mm model, **c** PAT 5-mm model

Prior to developing RM for grading prediction, we conducted a three-step radiomics feature screening process. This process involved repeatability analysis, *t*-test or *U*-test, and Spearman’s analysis (Appendix S6). To further refine our RMs, we used the LASSO method and retained a total of 16 optimal radiomics features for each ROI type. The specific Radscore formulas for these three RMs are shown in Fig. 3.

### Radiomics model analysis

In the three cohorts, the peritumoral areas of the tumor (PAT) 5-mm models (RM_5_) exhibited exceptional performance in predicting grading, achieving AUC values of 0.80, 0.80, and 0.90, respectively (Table 3). These values surpassed those of the internal area of the tumor (IAT) model (RM) (0.79, 0.66, 0.70) and the PAT 3-mm model (RM_3_) (0.80, 0.77, 0.83). DeLong’s test confirmed that the AUC value of the RM_5_ and RM_3_ models significantly differed from the RM models in both the internal validation (0.80 vs. 0.66, *p* = 0.01; 0.77 vs. 0.66, *p* = 0.03) and external validation (0.90 vs. 0.70, *p* = 0.01; 0.80 vs. 0.70, *p* = 0.04), but the difference between the RM_5_ and RM_3_ was not statistically significant in both of the internal validations (*p* > 0.05). The Wilcoxon test revealed that the RM_5_-gained Radscore was significantly higher for the high-grade patients than for the low-grade patients in all datasets (*p* < 0.05) (Supplemental Fig. 1). Consequently, we used the radiomics features of the RM_5_ model to construct the nomogram for predicting pathological grades.

**Table 3:** Prediction performance of the established models in the training set, internal validation set, and external validation set

	Model	AUC (95% CI)	Acc	Sen	Spe	PPV	NPV	Pre	Rec	F1	Thr
TS	RM	0.79 (0.72–0.85)	0.69	0.77	0.67	0.33	0.94	0.32	0.77	0.46	0.18
	RM_3_	0.80 (0.73–0.87)	0.82	0.65	0.86	0.48	0.92	0.48	0.65	0.55	0.28
	RM_5_	0.80 (0.73–0.87)	0.67	0.85	0.63	0.32	0.96	0.32	0.85	0.46	0.13
	CM	0.61 (0.55–0.68)	0.82	0.25	1.00	0.67	0.87	0.67	0.25	0.36	0.66
	FM	0.93 (0.89–0.98)	0.92	0.92	0.92	0.70	0.98	0.70	0.92	0.80	0.14
IVS	RM	0.66 (0.50–0.82)	0.68	0.71	0.68	0.35	0.95	0.35	0.71	0.47	0.14
	RM_3_	0.77 (0.65–0.89)	0.70	0.93	0.66	0.39	0.97	0.39	0.93	0.55	0.13
	RM_5_	0.80 (0.65–0.95)	0.72	0.86	0.68	0.40	0.95	0.40	0.86	0.55	0.12
	CM	0.57 (0.48–0.67)	0.83	0.14	1.00	1.00	0.83	1.00	0.14	0.25	0.66
	FM	0.78 (0.63–0.92)	0.80	0.64	0.84	0.50	0.91	0.50	0.64	0.56	0.20
EVS	RM	0.70 (0.52–0.89)	0.75	0.46	0.88	0.67	0.77	0.67	0.46	0.55	0.24
	RM_3_	0.83 (0.66–0.99)	0.80	0.85	0.78	0.65	0.91	0.65	0.84	0.73	0.15
	RM_5_	0.90 (0.80–0.99)	0.80	1.00	0.70	0.62	1.00	0.62	1.00	0.77	0.09
	CM	0.61 (0.50–0.73)	0.75	0.23	1.00	1.00	0.73	1.00	0.23	0.38	0.66
	FM	0.71 (0.50–0.92)	0.78	0.62	0.85	0.67	0.82	0.67	0.62	0.64	0.31

*TS* the training set, *IVS* internal validation set, *EVS* external validation set, *RM* radiomics model, *RM*_*3*_ radiomics model based on intratumoral and peritumoral 3 mm regional features, *RM*_*5*_ radiomics model based on intratumoral and peritumoral 5 mm regional features, *CM* clinical model, *FM* fusion model incorporated both the intratumoral and peritumoral 5 mm regional features and clinical factors, *Acc* accuracy, *Sen* sensitivity, *Spe* specificity, *PPV* positive predictive value, *NPV* negative predictive value, *Pre* prevalence, *Rec* recall, *F1* F1 score, *Thr* threshold

### Radiomics nomogram construction and survival analysis for OS

**Fig. 4:**
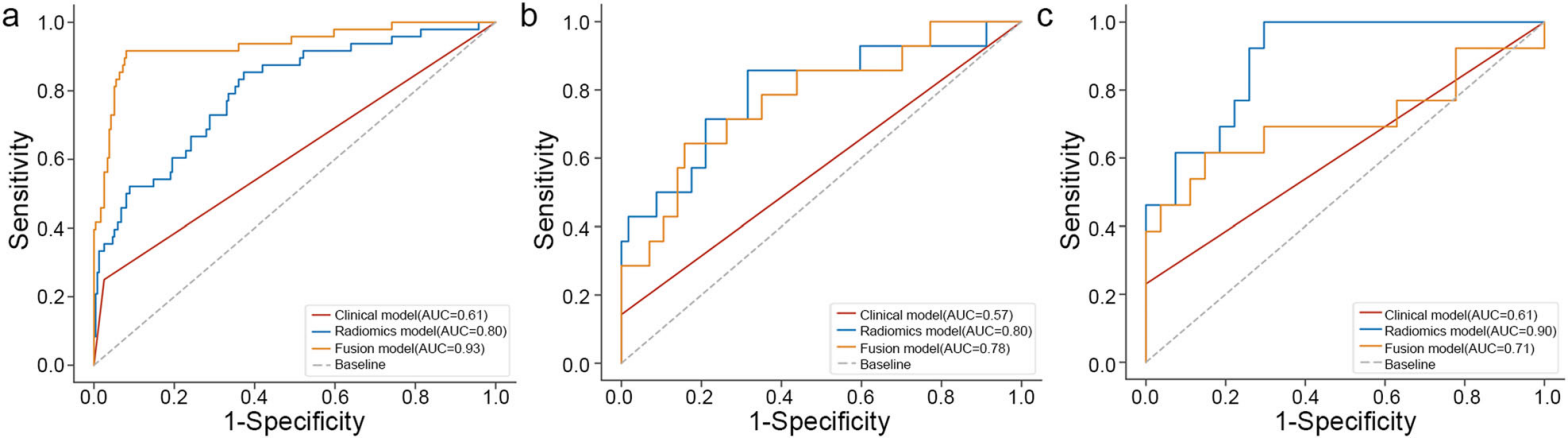
ROC comparison of the clinical model, radiomics model, and fusion model in (**a**) the training set, (**b**) internal validation set, and (**c**) external validation set

**Fig. 5:**
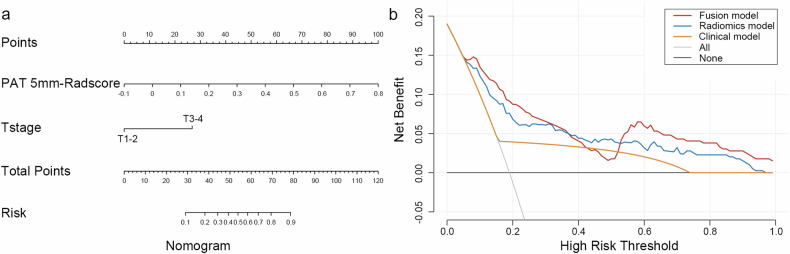
Demonstration of the nomogram (fusion model). **a** A fusion model incorporating the PAT 5-mm-gained Radscore and T stage was constructed. **b** The decision curve analysis of the clinical model, radiomics model, and fusion model based on the whole cohort. Radiomics model based on the PAT 5-mm region

**Fig. 6:**
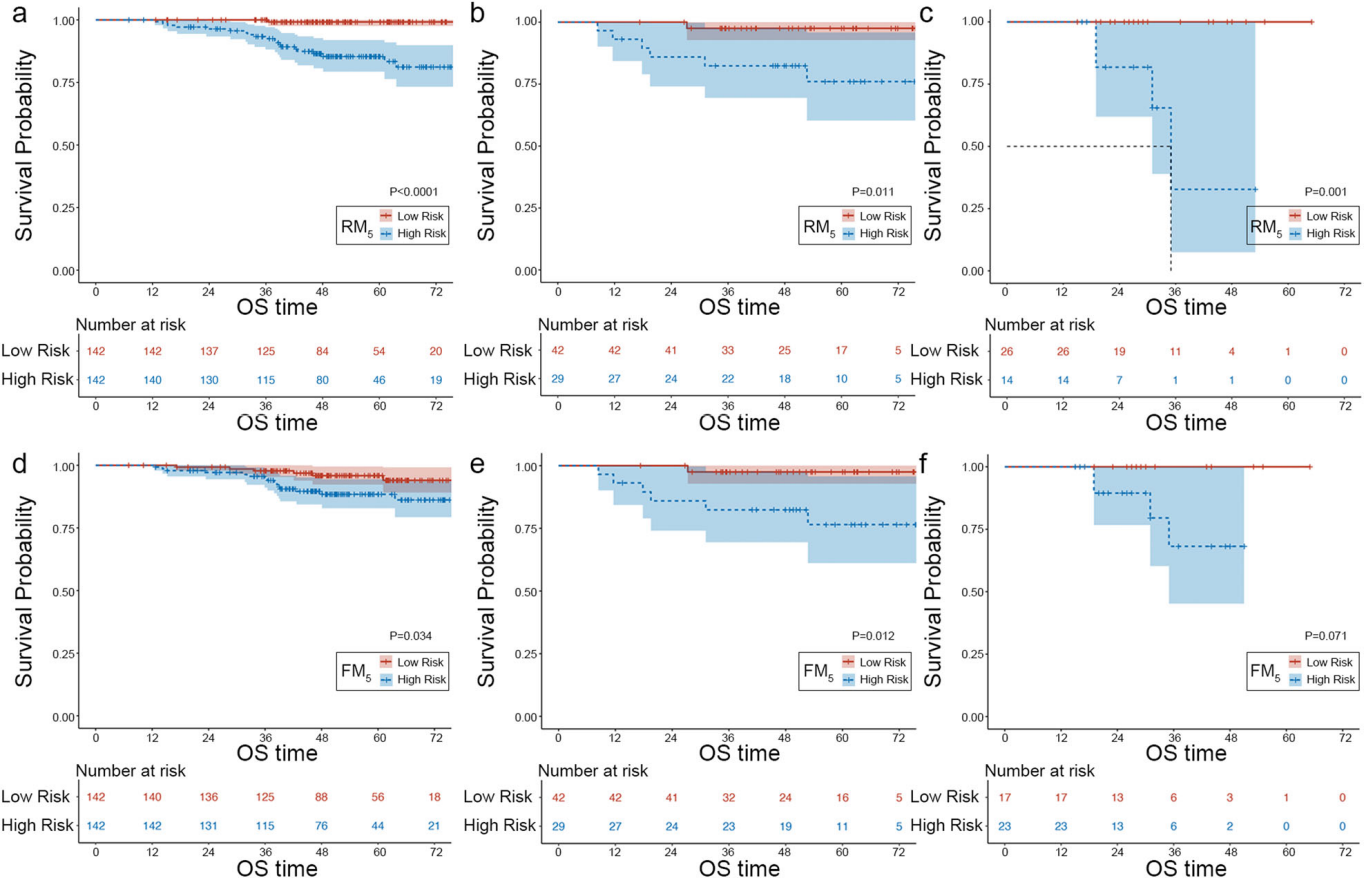
Kaplan–Meier survival curves were generated for the RM_5_ and FM_5_ models in the training (**a**, **d**), internal validation (**b**, **e**), and external validation cohorts (**c**, **f**). RM_5_, Radiomics model based on the PAT 5-mm region; FM, a fusion model incorporating the PAT 5-mm-gained Radscore and T stage

A multivariate logistic regression analysis was performed to determine the factors that independently predicted ccRCC grade, namely the radiomics signature and T stage (Table 2). To create an FM model, a nomogram was developed that incorporated both the PAT 5-mm radiomics signature and the T stage. The ROC curves of the PAT 5-mm RM (RM_5_), CM, and FM model were compared to assess their effectiveness in predicting the grade of ccRCC in the three cohorts (Fig. 4 and Table 3). The AUC was higher in the RM model than in the CM in the internal validation cohort (0.80 vs. 0.57, *p* = 0.003) and external validation cohort (0.90 vs. 0.61, *p* < 0.001). The nomogram’s AUC was higher than the CM’s AUC in the internal validation cohort (0.78 vs. 0.57, *p* = 0.005) and external validation cohort (0.71 vs. 0.61, *p* = 0.332). The AUC of the nomogram was not improved relative to the RM_5_ in the internal validation cohort (0.78 vs. 0.80, *p* = 0.422) and in the external validation cohort (0.71 vs. 0.90, *p* = 0.033). Additionally, the DCA analysis demonstrated that both the FM and RM_5_ models had a higher net benefit in predicting grading than that of the CM in the total dataset (Fig. 5). Finally, the Kaplan–Meier survival curves revealed that OS, as determined by the PAT 5-mm Radscore-predicted and nomogram-predicted grading, was significantly worse for the high-grade patients than for the low-grade patients in all cohorts except for the survival curve of the nomogram-predicted score in the external validation set (Fig. 6).

### Pathological characteristics and association with radiomics features

We detected cases with pathological features like perirenal or sinus fat invasion in peritumoral tissues and examined their relationship with radiomics features chosen from the PAT 5-mm model. Our analysis revealed that a significant number of these peritumoral parameters (7 out of 16) are connected to invasive biological behaviors (*p* < 0.05) (See Supplemental Fig. 2).

## Discussion

The study findings indicate that radiomics features, particularly peritumoral and tumoral signatures, serve as more precise indicators for grading and predicting survival. Additionally, the study demonstrated that the RM and nomogram performed better than the CM.

Invasion of tumor cells often disrupts the normal structure of the surrounding parenchymal tissues and leads to alterations in the peritumoral microenvironment. Unfortunately, the significance of this peritumoral microenvironment is sometimes overlooked in research that primarily focuses on the internal aspects of the tumor [20, 24]. Conventional imaging techniques struggle to accurately depict the microenvironment around a tumor. However, peritumoral characteristics offer the potential for quantitatively analyzing the heterogeneity of the microenvironment [25, 26]. In the present study, we constructed RMs for IAT and PAT with the goal of predicting the grading of ccRCC and investigating its association with survival prognosis. Our results revealed that the PAT model (PAT 3 mm and PAT 5 mm) had higher predictive values than that of the IAT model. This finding confirms that incorporating incremental information from both the internal tumor components and peritumoral features into a combined model can significantly enhance predictive performance. Interestingly, our findings align with those of previous reports showing that all peritumoral-feature models yielded grading performance superior to that based on features solely within the tumor [22]. Pathological research has demonstrated the crucial role of the peritumoral microenvironment in evaluating tumor invasiveness [27]. Pathological grading is correlated with tumor invasiveness [28]. Tumors of higher grades show increased invasiveness, leading to the invasion of surrounding tissues. As a consequence, the tumor’s surrounding environment undergoes heterogeneous alterations. Consequently, the evaluation of intratumoral grading heavily relies on peritumoral characteristics, which capture the heterogeneity within the tumor and its surroundings [17]. Our study revealed an association between the histological characteristics of perirenal fat invasion and radiomics features. This finding provides evidence that the radiomics features obtained from the PAT region can reflect the biological behavior of tumors. Therefore, when delineating ROIs, it is important not to overlook areas within the tumor and those outside the tumor, as this is associated with tumor proliferation and heterogeneity. Hence, the PAT model developed in this study automatically extends to the surrounding region based on the delineation of the IAT, not only eliminating the need for time-consuming operations to remove the internal tumor mask but also improving the clinical workflow.

The analysis of feature selection revealed that the first-order texture feature “ exponential_firstorder_Kurtosis,” which was obtained by exponentiating the “firstorder_Kurtosis,” had the strongest correlation with the grade in the peritumoral 5 mm area. The relationship between kurtosis and tumors can be inferred from the morphological characteristics of tumors [29, 30]. A higher kurtosis value indicates greater cell density within the tumor, implying a uniform and densely structured tumor. Kurtosis is frequently employed in radiomics to assess tumor heterogeneity [31]. Higher values of kurtosis may indicate increased tumor heterogeneity, which has a potential correlation with tumor malignancy. Our research supports this relationship, as we have discovered a close association between higher kurtosis and tumor malignancy grade. “Entropy” and “JointEntropy” are two terms that measure the uncertainty or disorder in a random variable or joint probability distribution, respectively. Higher values indicate greater heterogeneity [32]. Previous studies [33, 34] have also highlighted the close association between entropy and tumor invasiveness, which aligns with the findings of our research. From a pathologic perspective, the grades of ccRCC are determined by nuclear diameter, nuclear shape, and nucleoli. A higher grade is characterized by a larger nuclear diameter, a more irregular nuclear shape, and greater irregularity in the arrangement of histological internal components in pathological sections. “Busyness” is an additional crucial feature in the PAT 5 mm model, which measures the intensity change between a pixel and its neighboring pixels. A high value for busyness indicates a “busy” image with rapid changes in CT intensity between a pixel and its neighboring pixels, often signifying the rate of tumor growth or progression. Busyness is linked to tumor prognosis [35, 36]. Our study indicates a positive correlation between this parameter and tumor grade, as well as a close association with perirenal or perirenal sinus fat invasion. To summarize, quantitative analysis of radiomics features is a non-intrusive strategy to better understand the biological properties of tumors. This strategy can provide a substantial foundation for achieving personalized precision treatment of ccRCC.

We then analyzed the clinical risk factors, which have the potential to offer supplemental data and enhance the accuracy of the predictive model [37, 38]. Previous research has prioritized the examination of radiomic characteristics while neglecting clinical risk factors [9–16]. In this investigation, we performed multivariable logistic regression analysis and identified the T stage as an independent predictive factor. This finding aligns with the results of Zheng et al [17] who demonstrated a strong association between T stage and renal cancer grade. The AUC of the nomogram was improved relative to that of the RM in the training set (0.93 vs. 0.80). However, there was no improvement observed in the internal validation (0.78 vs. 0.80) and external validation (0.61 vs. 0.71), indicating that clinical risk factors may not have a strong predictive value for grade. Nevertheless, the nomogram-gained score was able to predict OS, highlighting the importance of both the RM and the nomogram in clinical applications.

Our study had several limitations. Initially, this study was conducted retrospectively and did not encompass chromophobe or papillary tumors and those who will not have nephrectomy, thus potentially introducing selection bias. We collected data on a limited number of cases of chromophobe and papillary RCC (approximately 20–30 cases). The small sample size could potentially impact the reliability of the model and statistical results, affecting the statistical power. In the future, we will expand the sample size and add more cases of other types. To assess the robustness and consistency of our models, we used a dataset from a different institution to evaluate their effectiveness. However, it is important to note that the predictive accuracy of the external validation cohort may be limited by the small sample size and relatively short follow-up period. Consequently, further validation through additional studies incorporating larger sample sizes is a necessary step for future research.

To summarize, the performance of the PAT RM outperformed the IAT model for predicting ccRCC grade before surgery. Additionally, both nomograms and RMs improved predictive accuracy and survival prediction. This advancement can help in adjusting treatment strategies promptly and establishing prognostic risk stratification.

### Supplementary information

ELECTRONIC SUPPLEMENTARY MATERIAL

## Data Availability

The datasets generated and analyzed during the current study are not publicly available but are available from the corresponding author on reasonable request.

## Abbreviations

AUC: Area under the curve
ccRCC: Clear cell renal cell carcinoma
CM: Clinical model
DCA: Decision curve analysis
FM: Fusion model
IAT: Internal area of the tumor
ICC: Intraclass correlation coefficient
ISUP: International Society of Urological Pathology
LASSO: Least absolute shrinkage and selection operator
OS: Overall survival
PAT: Peritumoral areas of the tumor
RM: Radiomics model
Radscore: Radiomics scores
ROI: Regions of interest
RCC: Renal cell carcinoma
WHO: World Health Organization
