# Supplementary material for: Radiomics predict the WHO/ISUP nuclear grade and survival in clear cell renal cell carcinoma
Source: Insights Imaging. 2024 Jul 12;15:175. doi: 10.1186/s13244-024-01739-z (PMC11239644; doi:10.1186/s13244-024-01739-z)

Radiomics predict the WHO/ISUP nuclear grade and survival in clear cell renal cell carcinoma

ELECTRONIC SUPPLEMENTARY MATERIAL

Appendix S1

The following inclusion criteria were applied: (1) underwent preoperative contrast-enhanced CT, (2) histological subtype of ccRCC, and (3) complete image and clinical information was available. The following exclusion criteria were applied: (1) compromised CT image quality (n= 24), (2) previous preoperative therapy (targeted therapy, etc.) (n= 19), and (3) incomplete image and clinical information (n= 28).

Appendix S2

Our hospital uses the following multidetector CT scanners: GE Lightspeed 64 (Ge Healthcare Chicago, Il, USA), Siemens Sensation 16 (Siemens Healthineers, Erlangen, Germany) and Siemens Definition 64 (Siemens Healthineers). The parameters were as follows: tube voltage 120 kVp, tube current 150–200 mA, Pixel size, 0.4-1.0mm, slice thickness 5 mm, interval thickness 5 mm, and nonionic contrast media iomeperol at a rate of 3.0 ml/s with a dose of 1.3 ml/kg. A 100-Hu threshold of the abdominal aorta at the celiac artery was taken as the baseline, then the arterial phase was examined 15-30 s after the unenhanced phase.

Appendix S3

The regions of interest (ROIs) of RCC were identified by two experienced radiologists. The internal area of the tumor (IAT) refers to the entire tumor volume delineated layer by layer along the tumor border including necrotic, cystic change and hemorrhagic areas, but excluding normal renal tissue, perinephric and sinus fat. Previous research [1] has shown that a 5-mm region surrounding the tumor could provide valuable information about the heterogeneity of ccRCC. According to this study, to obtain the peritumoral ROIs, the IAT was uniformly expanded, resulting in the acquisition of the intratumoral and peritumoral area of the tumor (PAT), which included the PAT 3-mm and PAT 5-mm regions. In this study, the segmentation of PAT was an automatically expanding method, meaning the PAT would contain some normal renal tissue, perinephric, sinus fat, or microvessel. The main advantage of this automatic process was to reduce manual intervention of PAT segmentation and to ensure the consistency and reproducibility. However, due to the limited size of the perirenal space, in the case of larger tumors, expanding the region of interest (ROI) by 3mm or 5mm from the tumor's inner boundary may cause the ROI to extend beyond the abdominal wall or into areas outside the abdominal cavity. As a result, after the automatic delineation process, it is crucial to thoroughly review and manually modify the ROI area that extends beyond the abdominal wall, ensuring that it remains confined within the abdominal cavity.

Appendix S4

To obtain more high-throughput features, wavelet, gradient, logarithm, exponent, square, square root and Laplacian of Gaussian (LoG) filters were used to transform the normalized CT images. Then, the first-order features and texture features, including the gray-level size zone matrix (GLSZM), graylevel co-occurrence matrix (GLCM), neighborhood graytone difference matrix (NGTDM), gray-level dependence matrix (GLDM) and gray-level run length matrix (GLRLM), were extracted from the transformed images. 1834 features were extracted for each type of ROI, and each patient had a total of 3 types of ROI, totaling 5502 features.

Appendix S5

To assess the reproducibility of these features, a set of 30 CT images was randomly selected and evaluated. Interobserver repeatability was determined by comparing the IAT, PAT 3-mm, and PAT 5-mm ROI delineations performed by Radiologist 1 (L.X.) and Radiologist 2 (G.Y.) within the same timeframe. Additionally, to evaluate intraobserver repeatability, Radiologist 1 conducted a second delineation 1 month later. Throughout the evaluation process, both radiologists remained unaware of the pathological findings. The intraclass correlation coefficient (ICC), where an ICC value exceeding 0.75 indicated a high level of consistency, was used to measure the extent of agreement between the assessments.

Appendix S6

A total of 1834 radiomics features were extracted from the ROIs of the IAT, PAT 3 mm, and PAT 5 mm. The reproducibility of these features was excellent, with 86.0% (1579/1834) of IAT features, 92.4% (1694/1834) of PAT 3-mm features, and 91.0% (1669/1834) of PAT 5-mm features demonstrating high consistency. Both interobserver and intraobserver ICC values exceeded 0.75, indicating strong agreement. Either the *t*-test or *U*-test identified 1022 features selected for the IAT ROIs, 971 features selected for the PAT 3-mm ROIs, and 947 features selected for the PAT 5-mm ROIs. Additionally, Spearman's analysis selected 191 features for the IAT ROIs, 183 features for the PAT 3-mm ROIs, and 180 features for the PAT 5-mm ROIs.

Reference

1. Zhou Z, Qian X, Hu J et al (2021) CT-based peritumoral radiomics signatures for malignancy grading of clear cell renal cell carcinoma. Abdom Radiol (NY) 46: 2690-2698.

Supplementary Figures

Figure S1. The Wilcoxon test of the PAT 5-mm-gained Radscore for low and high grades in the (a) training set, (b) internal validation set, and (c) external validation set.

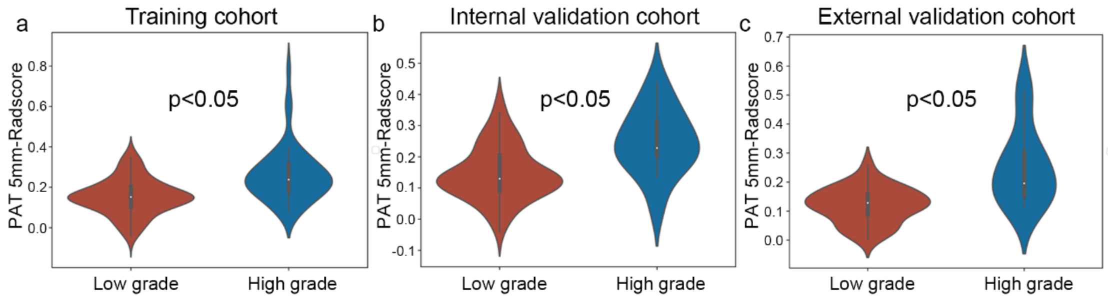

Figure S2. The Wilcoxon test of the PAT 5-mm-gained radiomicis parameters for identification of perirenal and sinus fat invasion on whole set.
***Significant results.

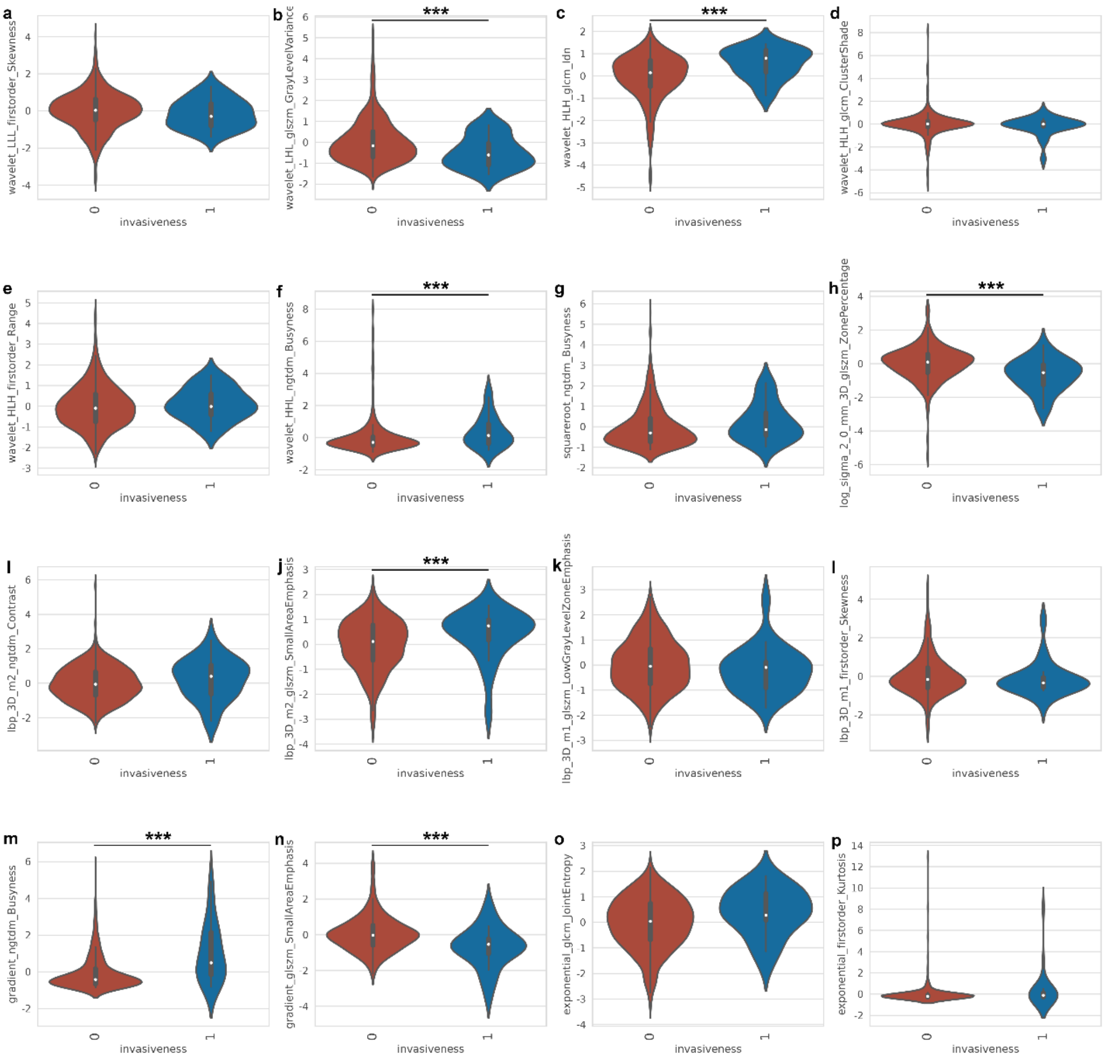

Supplement: Supplementary file 1 — ELECTRONIC SUPPLEMENTARY MATERIAL [file 13244_2024_1739_MOESM1_ESM.pdf]
